# Nontuberculous Mycobacterial Disease in Solid-Organ Transplant Recipients and the General Population

**DOI:** 10.1001/jamanetworkopen.2025.31563

**Published:** 2025-09-12

**Authors:** Seyed M. Hosseini-Moghaddam, Daniel Fridman, Samantha S. M. Drover, Theodore K. Marras, Sarah K. Brode, Frances B. Jamieson, Shahid Husain, Jeffrey C. Kwong

**Affiliations:** 1 ICES, Toronto, Ontario, Canada; 2 Transplant-Oncology Infectious Diseases, Ajmera Transplant Program, University Health Network, Toronto, Ontario, Canada; 3 Division of Infectious Diseases, Department of Medicine, University of Toronto, Toronto, Ontario, Canada; 4 Division of Respirology, Department of Medicine, University of Toronto, Toronto, Ontario, Canada; 5 University Health Network, Toronto, Ontario, Canada; 6 Public Health Ontario, Toronto, Ontario, Canada; 7 Department of Laboratory Medicine and Pathobiology, University of Toronto, Toronto, Ontario, Canada; 8 Department of Family and Community Medicine, University of Toronto, Toronto, Ontario, Canada; 9 Dalla Lana School of Public Health, University of Toronto, Toronto, Ontario, Canada; 10 Centre for Vaccine Preventable Diseases, University of Toronto, Toronto, Ontario, Canada

## Abstract

**Importance:**

Single-center studies suggest that solid-organ transplant recipients (SOTRs) with nontuberculous mycobacterial disease (NTM-D) face increased mortality, particularly in the presence of comorbidities such as chronic lung disease. Large-scale studies are needed to quantify the risk of NTM-D and its impact on mortality in this population.

**Objective:**

To compare the risk of NTM-D between the general population and SOTRs, stratified by lung and nonlung transplants, and to assess whether NTM-D is associated with increased mortality risk in SOTRs.

**Design, Setting, and Participants:**

This population-based cohort study was conducted in Ontario, Canada, from April 1, 2002, to December 31, 2018. Adult SOTRs were matched 1:10 with general population controls by age, sex, and region. Analyses were conducted from January 2024 to March 2025.

**Exposure:**

Solid-organ transplantation.

**Main Outcomes and Measures:**

The primary outcome was NTM-D, defined as isolation of NTM from blood, tissues, or respiratory samples (≥2 sputum cultures with the same species or 1 bronchoscopic or lung biopsy culture). The secondary outcome was all-cause mortality in the SOTR cohort at 1 year and by the end of follow-up (March 31, 2021). Cox proportional hazard models were used to estimate the risk of outcomes.

**Results:**

The study included 138 175 individuals (49 611 female [35.9%]; mean [SD] age, 51.87 [12.99] years), comprising 12 564 SOTRs (7674 kidney, 2419 liver, 1257 lung, 584 heart, 563 kidney-pancreas, and 67 multiorgan recipients) and 125 611 matched controls. During the study period, 368 SOTRs (2.92%) and 127 controls (0.10%) developed NTM-D. Receipt of a lung transplant (adjusted hazard ratio [aHR], 177.34; 95% CI, 79.65-394.82) or other solid-organ transplant (aHR, 8.89; 95% CI, 5.90-13.40) were both associated with increased risk of NTM-D compared with controls. NTM-D by *Mycobacterium avium *complex (MAC) and rapidly growing mycobacteria (RGM) were associated with significant long-term mortality in lung SOTRs (MAC aHR, 1.66; 95% CI, 1.35-2.04; RGM aHR, 2.33; 95% CI, 1.59-3.39) and nonlung SOTRs (MAC aHR, 2.12; 95% CI, 1.36-3.29; RGM aHR, 2.25; 95% CI, 1.07-4.74).

**Conclusions and Relevance:**

In this cohort study of SOTRs and the general population, both lung transplantation and other solid-organ transplantation were associated with a significantly elevated risk of NTM-D, which was linked to a higher mortality risk. These findings highlight the need for preventive and screening strategies.

## Key Points

**Question:**

Are solid-organ transplant recipients (SOTRs) at increased risk of nontuberculous mycobacterial disease (NTM-D) compared with the general population?

**Findings:**

In this population-based cohort study of 138 175 individuals in Ontario, lung and nonlung SOTRs had a significantly higher risk of developing NTM-D and faced increased mortality after diagnosis compared with matched individuals from the general population.

**Meaning:**

These findings suggest that preventive and screening strategies are needed to reduce the risk of NTM-D in both lung and nonlung SOTRs.

## Teaser

This cohort study examines whether nontuberculous mycobacterial disease is associated with increased mortality risk among solid-organ transplant recipients.

## Introduction

Nontuberculous mycobacteria (NTM), once considered harmless saprophytes, have been recognized as human pathogens since the 1980s.^1^ These diverse acid-fast microorganisms are found in water, soil, food products, and both domestic and wild animals,^1,2,3,4,5,6^ and can infect lymph nodes, skin, lungs, and the bloodstream.^3^

NTM infection and NTM disease (NTM-D) have increased globally over the past decade among both immunocompromised and immunocompetent individuals.^7,8,9,10^ This increase may be associated with population aging, climate change, ecosystem disruption, mixing of ocean water with fresh water and water with soil, plumbing system and showerhead biofilms, increasing incidence of chronic lung diseases known to be associated with NTM-D (eg, chronic obstructive pulmonary disease and bronchiectasis), and advances in diagnostic methods.^11,12,13,14,15^ The prevalence of NTM exposure varies depending on geographic location, with warmer and more humid regions exhibiting higher rates, since these organisms thrive in such climates.^11,15^

Immunocompromised patients, particularly solid-organ transplant recipients (SOTRs), appear to have a high frequency of NTM infection.^16^ Single-center studies have reported varying rates of NTM-D in SOTRs.^16,17^ No comparative studies have evaluated the risks faced by SOTRs compared with the general population. This knowledge gap is important, especially given the simultaneous influence of various factors, including age, underlying lung diseases, immunocompromising conditions, and geographic differences contributing to NTM infections.

The other issue is the controversial association between NTM-D and mortality in SOTRs. Aside from infection with rapidly growing mycobacteria (RGM), NTM-D is not typically associated with acute illness. Although some studies have demonstrated a possible association between NTM-D and mortality in lung transplant recipients,^18^ this association has not been investigated for nonlung SOTRs and slowly growing NTM. Some studies showed that SOTRs with NTM-D are not at increased risk of death compared with those without NTM-D.^19,20,21^ With few exceptions,^22^ most available studies are single-center observational analyses with small sample sizes and limited follow-up durations after NTM-D diagnosis, making it difficult to accurately assess the impact of NTM-D on patient outcomes.^21,23^

The limited understanding of the epidemiology and impact of NTM-D in SOTRs has led to uncertainty regarding the development of appropriate prevention and surveillance strategies. We investigated the risk of NTM-D in SOTRs compared with the general population and examined mortality among SOTRs with NTM-D vs those without mycobacterial infection. All analyses were stratified by lung and nonlung transplant recipients.

## Methods

### Study Design, Setting, and Population

We conducted a population-based cohort study of residents in Ontario, Canada, comparing SOTRs with the general population. Each SOTR was matched with 10 non-SOTR controls according to age, sex, and geographic area of residence, which was defined by the Forward Sortation Area (the first 3 characters of their postal code). We obtained ethics approval from University Health Network and Public Health Ontario, with a waiver of informed consent owing to the retrospective use of deidentified administrative data. Our findings were reported in accordance with the Strengthening the Reporting of Observational Studies in Epidemiology (STROBE) reporting guidelines.

We included all individuals aged 18 years or older who underwent organ transplant in Ontario between April 1, 2002, and December 31, 2018 (eFigure in Supplement 1). Individuals with missing or invalid age or sex, nonresidents of Ontario, or those who died on or before the index date were excluded. We established a matched retrospective cohort from the general population, considering year of birth, sex, and geographic area of residence. The index date for SOTRs was their transplant date, whereas the matched control from the general population had the same index date as their corresponding case. To assess mortality outcomes, the observation period was extended to March 31, 2021.

### Exposure and Outcomes

We used the Canadian Organ Replacement Register to identify individuals who underwent solid-organ transplant in Ontario during the study period. Our primary outcome of interest was a culture-proven NTM-D diagnosis following the index date. For pulmonary NTM-D, the diagnosis was based on mycobacterial culture data, which included detecting the same NTM species in 2 or more sputum cultures or at least 1 bronchoalveolar lavage or pulmonary biopsy specimen.^24,25,26,27^ For nonpulmonary NTM-D, the diagnosis was limited to those with isolation of NTM from tissue specimens or blood cultures. In the case of recurrent NTM-D, we only included the first NTM-D episode as a positive outcome. We categorized a group of NTM infections under 2 categories: *Mycobacterium avium* complex (MAC) which in our cohort included *M avium, M intracellulare *group (including *M chimaera*), and *M marseillense*, and RGM, which included *M fortuitum* group, *M chelonae*,* M abscessus*,* M mucogenicum-phocaicum* group, and *M neoaurum. M xenopi* and *M lentiflavum* were analyzed separately because of their higher frequency in the cohort. As a secondary outcome, we assessed all-cause mortality among SOTRs, comparing those who developed NTM-D and those without NTM infection.

### Data Sources and Definitions

Using unique encoded identifiers, we linked Canadian Organ Replacement Register data with mycobacterial culture results from Public Health Ontario and health administrative databases. Public Health Ontario captures more than 95% of NTM isolates and 100% of *M tuberculosis* isolates in Ontario.^28^ Definitions and data sources for variables and comorbidities can be found in eTable 1 in Supplement 1. The data were analyzed at ICES (formerly the Institute for Clinical Evaluative Sciences), a not-for-profit research institute. Data use was authorized under Section 45 of Ontario’s Personal Health Information Protection Act.

### Covariates

We used a variety of health-related administrative databases to identify various comorbidities. From Ontario’s Registered Persons Database, we gathered information on age, sex, residential postal code, and date of death. Using the residential postal code, we determined each individual’s public health unit of residence. Frailty was assessed using a validated acute care contextual frailty score, ranging from 1 (least frail) to 8 (most frail).^29^

### Statistical Analysis

We used standardized differences to compare baseline characteristics and Cox proportional hazards models, stratified by lung and nonlung SOTRs, to identify NTM-D risk factors by comparing SOTRs with matched controls from the general population. Sample sizes less than 6 were suppressed to preserve anonymity. We calculated 1-year and long-term mortality rates for SOTRs with NTM-D vs those without any mycobacterial infection, deriving rate ratios (RRs) with 95% CIs. Mortality risk was estimated by comparing lung and nonlung SOTRs with NTM-D to recipients of the same organ type who remained free of mycobacterial infection during the study period. In this comparison, we adjusted for several potential confounders, including comorbidities, frailty score, immigration status, and urban vs rural residence. Because of presumed elevated risk in addition to surveillance bias for pulmonary NTM-D among lung transplant recipients, secondary analyses investigated lung vs nonlung transplant recipients separately. Statistical significance was defined as a 2-sided *P* < .05 and a 95% CI excluding 1.0 for hazard ratios (HRs) and RRs. Analyses were performed using SAS statistical software version 9.4 (SAS Institute) from January 2024 to March 2025.

## Results

### Study Population

A total of 12 564 patients (4510 female [35.9%]; mean [SD] age, 51.87 [12.99] years) received organ transplants during the study interval, including 7674 kidney (61.8%), 2419 liver (19.3%), 1257 lung (10.0%), 584 heart (4.6%), 563 kidney-pancreas (4.5%), and 67 multiorgan (0.5%) allografts. We included 125 611 matched individuals (45 101 female [35.9%]; mean [SD] age, 51.87 [12.99] years) from the general population (Table 1).

**Table 1.** Baseline Comparison of SOTRs and Non-SOTRs

Characteristic	Patients, No. (%)			*P* value
	SOTR (n = 12 564)	Non-SOTR (n = 125 611	Total (N = 138 175)	
Sex				
Female	4510 (35.9)	45 101 (35.9)	49 611 (35.9)	.98
Male	8054 (64.1)	80 510 (64.1)	88 564 (64.1)	
Age, y				
Mean (SD)	51.87 (12.99)	51.87 (12.99)	51.87 (12.99)	.99
Age group				
18-24	356 (2.8)	3572 (2.8)	3928 (2.8)	
25-34	1107 (8.8)	11 065 (8.8)	12 172 (8.8)	
35-44	1877 (14.9)	18 752 (14.9)	20 629 (14.9)	
45-54	3180 (25.3)	31 806 (25.3)	34 986 (25.3)	
55-64	3906 (31.1)	39 037 (31.1)	42 943 (31.1)	
65-74	1922 (15.3)	19 230 (15.3)	21 152 (15.3)	
75-84	175 (1.4)	1752 (1.4)	1927 (1.4)	
≥85	41 (0.3)	397 (0.3)	438 (0.3)	
Rural residential area	1357 (10.8)	13 674 (10.9)	15 031 (10.9)	.25
Missing	11 (0.1)	181 (0.1)	192 (0.1)	
Immigrant	1772 (14.1)	23 153 (18.4)	24 925 (18.0)	<.001
Chronic kidney disease	9195 (73.2)	1933 (1.5)	11 128 (8.1)	<.001
Chronic heart disease	3240 (25.8)	5421 (4.3)	8661 (6.3)	<.001
Advanced liver disease	2955 (23.5)	751 (0.6)	3706 (2.7)	<.001
Chronic lung disease	3547 (28.2)	18 740 (14.9)	22 287 (16.1)	<.001
Stroke or transient ischemic attack	590 (4.7)	1990 (1.6)	2580 (1.9)	<.001
Hypertension	9788 (77.9)	33 578 (26.7)	43 366 (31.4)	<.001
Diabetes	5193 (41.3)	14 187 (11.3)	19 380 (14.0)	<.001
Inflammatory bowel disease	98 (0.8)	330 (0.3)	428 (0.3)	<.001
Rheumatoid arthritis	284 (2.3)	1054 (0.8)	1338 (1.0)	<.001
Dementia	40 (0.3)	580 (0.5)	620 (0.4)	<.001
Frailty Risk Score, mean (SD)	3.91 (4.89)	0.22 (1.34)	0.55 (2.22)	<.001

Abbreviation: SOTR, solid-organ transplant recipient.

### Primary Outcome

Among SOTRs, 405 NTM isolates were identified, including 37 NTM-D episodes involving 2 distinct isolates (eTable 2 in Supplement 1). Over the study period, 368 SOTRs (2.92%) and 127 non-SOTRs (0.10%) developed NTM-D. Compared with the matched non-SOTR cohort for each organ allograft, SOTRs experienced higher NTM-D incidence for lung (284 of 1257 SOTRs [22.59%] vs 21 of 12 560 non-SOTRs [0.17%]), heart (21 of 584 SOTRs [3.59%] vs <6 of 5840 non-SOTRs [≤0.10%]), kidney or kidney-pancreas (49 of 8237 SOTRs [0.59%] vs 76 of 76 721 non-SOTRs [0.09%]), and liver (14 of 2419 SOTRs [0.55%] vs 23 of 24 190 non-SOTRs [0.09%]) transplants. Extrapulmonary NTM-D was observed in 28 of 368 SOTRs (7.6%) and fewer than 6 of 127 individuals in the general population. MAC was the most frequent isolate in SOTRs and non-SOTRs (245 of 405 SOTRs [60.49%] vs 85 of 127 non-SOTRs [66.92%]), followed by *M xenopi* (73 of 405 SOTRs [18.02%] vs 16 of 127 non-SOTRs [12.59%]) and RGM (56 of 405 SOTRs [13.82%] vs 13 of 127 non-SOTRs [10.23%]).

In univariate analysis, both lung (HR, 281.57; 95% CI, 149.87-529.01) and nonlung (HR, 10.93; 95% CI, 8.00-14.93) SOTRs had significantly higher NTM-D risk compared with their respective control groups (eTable 3 in Supplement 1). In adjusted analysis, we further evaluated the impact of organ type by differentiating between lung and nonlung transplants while adjusting for other covariates. As shown in Table 2, both lung (adjusted HR [aHR], 177.34; 95% CI, 79.65-394.82) and nonlung transplantation (aHR, 8.89; 95% CI, 5.90-13.40) were significantly associated with increased NTM-D risk.

**Table 2.** Nontuberculous Mycobacterial Disease Risk in the Cohort, Adjusted Analysis Categorized Considering Lung and Nonlung Transplant

Organ type and variable	Adjusted HR (95% CI)
Nonlung transplant	
Transplantation	8.89 (5.90-13.40)
Chronic lung disease	2.58 (1.75-3.81)
Rheumatoid arthritis	4.23 (1.58-11.29)
Frailty Risk Score	1.01 (0.96-1.07)
Immigrant	1.15 (0.72-1.83)
Lung transplant	
Transplantation	177.34 (79.65-394.82)
Chronic lung disease	5.30 (2.41-11.62)
Rheumatoid arthritis	1.89 (0.09-36.13)
Frailty Risk Score	1.04 (0.90-1.20)
Immigrant	3.55 (1.29-9.78)

Abbreviation: HR, hazard ratio.

### Secondary Outcomes

#### One-Year Mortality in SOTR Cohort

The 1-year mortality incidence in the SOTR cohort was 62.1 deaths per 1000 person-years. In SOTRs with NTM-D, the 1-year mortality incidence was 96.7 deaths per 1000 person-years, whereas in SOTRs without NTM-D, it was 61.5 deaths per 1000 person-years (RR, 1.57; 95% CI, 1.02-2.42; *P* = .03). When comparing SOTRs with NTM-D caused by MAC with those without mycobacterial infection, 1-year mortality was significantly higher (RR, 1.84; 95% CI, 1.14-2.98; *P* = .01). In contrast, the difference was not statistically significant for NTM-D due to RGM (RR, 2.30; 95% CI, 0.86-6.16; *P* = .08).

In unadjusted analysis, NTM-D was significantly associated with an increased 1-year mortality risk (HR, 2.72; 95% CI, 1.78-4.17). However, in multivariable analysis (eTable 4 in Supplement 1), the association between 1-year mortality and NTM-D was not significant (aHR, 1.29; 95% CI, 0.83-2.01). In lung transplant recipients, no significant associations were detected between 1-year mortality and different NTM-D organisms (ie, MAC, RGM, *M xenopi, M lentiflavum,* and other NTM). However, in nonlung SOTRs, NTM-D secondary to MAC (aHR, 5.02; 95% CI, 1.59-15.83), RGM (aHR, 7.42; 95% CI, 1.04-53.03), and *M xenopi* (aHR, 10.25; 95% CI, 1.43-73.43) was significantly associated with 1-year mortality (Table 3).

**Table 3.** Stratified (Lung vs Nonlung SOTRs) Adjusted Cox Proportional Hazard Model Estimating the 1-Year and Long-Term Mortality Risk in SOTRs

Organ transplant type and variable	Adjusted HR (95% CI)	
	Mortality at 1 y	Long-term mortality
Nonlung transplant		
Age	1.03 (1.02-1.04)	1.05 (1.04-1.05)
Sex, female vs male	1.05 (0.87-1.26)	0.91 (0.84-0.98)
MAC	5.02 (1.59-15.83)	2.12 (1.36-3.29)
RGM	7.42 (1.04-53.03)	2.25 (1.07-4.74)
* M xenopi*	10.25 (1.43-73.43)	1.76 (0.73-4.22)
* M lentiflavum*	7.05 (0.98-50.67)	1.75 (0.44-7.02)
Other NTMs^a^	0.00	6.03 (2.25-16.16)
Chronic kidney disease	0.63 (0.49-0.81)	1.00 (0.89-1.12)
Chronic heart disease	1.82 (1.50-2.22)	1.43 (1.33-1.55)
Advanced liver disease	1.58 (1.25-2.01)	1.18 (1.06-1.31)
Chronic lung disease^b^	1.04 (0.85-1.26)	1.20 (1.11-1.30)
History of stroke or transient ischemic attack	0.80 (0.53-1.19)	1.30 (1.14-1.48)
Hypertension	0.64 (0.51-0.81)	0.96 (0.86-1.07)
Diabetes	1.19 (1.00-1.42)	1.34 (1.25-1.44)
Inflammatory bowel disease	1.98 (1.02-3.88)	1.79 (1.21-2.64)
Rheumatoid arthritis	0.90 (0.48-1.68)	1.22 (0.97-1.53)
Dementia	0.76 (0.19-3.08)	0.87 (0.52-1.45)
Frailty Risk Score	1.02 (1.01-1.04)	1.02 (1.02-1.03)
Rural residential status	1.04 (0.79-1.38)	0.98 (0.88-1.10)
Immigrant	1.03 (0.81-1.32)	0.79 (0.70-0.88)
Lung transplant		
Age	1.01 (1.00-1.02)	1.02 (1.01-1.03)
Sex, female vs male	0.80 (0.59-1.09)	0.83 (0.71-0.97)
MAC	0.94 (0.54-1.64)	1.66 (1.35-2.04)
RGM	1.45 (0.46-4.61)	2.33 (1.59-3.39)
* M xenopi*	0.19 (0.03-1.38)	0.97 (0.69-1.36)
* M lentiflavum*	1.15 (0.28-4.71)	1.06 (0.55-2.05)
Other NTMs^a^	0.00	NA
Chronic kidney disease	1.83 (1.17-2.88)	1.49 (1.14-1.95)
Chronic heart disease	1.37 (0.99-1.90)	1.17 (0.98-1.39)
Advanced liver disease	1.48 (0.75-2.92)	1.15 (0.77-1.72)
Chronic lung disease^b^	0.83 (0.58-1.20)	0.96 (0.78-1.18)
History of stroke, transient ischemic attack	0.92 (0.33-2.55)	1.14 (0.71-1.82)
Hypertension	1.01 (0.74-1.38)	0.91 (0.78-1.07)
Diabetes	0.95 (0.70-1.30)	1.19 (1.01-1.39)
Inflammatory bowel disease	3.49 (0.48-25.55)	4.10 (1.01-16.64)
Rheumatoid arthritis	2.01 (1.21-3.34)	1.17 (0.84-1.63)
Dementia	3.33 (0.44-25.13)	1.22 (0.30-4.96)
Frailty Risk Score	1.02 (0.98-1.07)	1.04 (1.02-1.06)
Rural residential status	1.21 (0.76-1.94)	1.04 (0.82-1.31)
Immigrant	2.06 (1.28-3.31)	1.09 (0.79-1.49)

Abbreviations: HR, hazard ratio; MAC, *Mycobacterium avium* complex; NA, not applicable; NTM, nontuberculous mycobacteria; RGM, rapidly growing mycobacteria; SOTR, solid-organ transplant recipient.

^a^ Other NTMs included nontuberculous mycobacterial diseases associated with *M farcinogenes*, *M genavense*, *M hemophilum*, *M llatzerense*, *M longobardum*, *M marinum*, and *M senegalense*. There was no other NTM in lung transplant patients.

^b^ The variable of chronic lung disease was present for all lung transplant recipients.

#### Mortality at the End of the Follow-Up Period

As of March 31, 2021, the mortality incidence in the SOTR cohort was 40.0 deaths per 1000 person-years, including a mortality incidence of 97.4 deaths per 1000 person-years in SOTRs with NTM-D and 38.8 deaths per 1000 person-years in SOTRs without NTM-D (RR, 2.51; 95% CI, 2.18-2.89; *P* < .001). The mortality incidence was significantly higher in SOTRs with NTM-D, compared with those without NTM infection, including patients with MAC (RR, 2.73; 95% CI, 2.31-3.23; *P* < .001), RGM (RR, 2.63; 95% CI, 1.90-3.64; *P* < .001), *M xenopi* (RR, 1.99; 95% CI, 1.47-2.70; *P* < .001), and *M lentiflavum* (RR, 2.29; 95% CI, 1.30-4.04; *P* = .003).

In unadjusted analysis, NTM-D was associated with a significant risk of long-term mortality (HR, 3.70; 95% CI, 3.21-4.26). eTable 4 in Supplement 1 presents adjusted Cox proportional hazard model estimating the long-term mortality risk in SOTRs. In this analysis, NTM-D was associated with a significant long-term mortality risk (aHR, 2.33; 95% CI, 2.00-2.71).

We observed significant mortality risk associated with MAC (aHR, 2.28; 95% CI, 1.89-2.73), RGM (aHR, 3.00; 95% CI, 2.14-4.19), and *M xenopi* (aHR, 1.39; 95% CI, 1.01-1.91), but not *M lentiflavum* (aHR, 1.63; 95% CI, 0.90-2.96) (eTable 5 in Supplement 1). In a stratified adjusted Cox proportional hazard model (Table 3), we separately estimated the long-term mortality risk in lung vs nonlung SOTRs. In this analysis, NTM-D caused by MAC was associated with an increased mortality risk in lung (aHR, 1.66; 95% CI, 1.35-2.04) and nonlung (aHR, 2.12; 95% CI, 1.36-3.29) SOTRs. Similarly, we observed an increased risk of mortality following NTM-D related to RGM in lung (aHR, 2.33; 95% CI, 1.59-3.39) and nonlung (aHR, 2.25; 95% CI, 1.07-4.74) SOTRs.

## Discussion

In this retrospective, provincewide cohort study, we found that SOTRs had a significantly higher risk of developing NTM-D compared with matched individuals from the general population, a risk that remained elevated after adjusting for comorbidities, particularly lung disease. The elevated risk was evident in both lung and nonlung SOTRs, suggesting that immunosuppression and other transplant-related factors substantially contribute to NTM-D susceptibility. Furthermore, NTM-D was associated with increased mortality in SOTRs, highlighting its clinical relevance as a potential marker of poor prognosis and the need for improved screening and management strategies. Further research is warranted to determine to what extent NTM-D may directly contribute to mortality or serves as an indicator of underlying vulnerability in this high-risk population.

Although small-scale studies have reported varying frequencies of NTM infection in SOTRs, to our knowledge, no prior study has directly compared the risk of NTM-D between SOTRs and the general population while accounting for key confounding factors, such as age, sex, geographic location, and particular comorbidities, all of which are known risk factors for NTM-D.^17,21,30^ Existing evidence on posttransplant NTM-D is limited by concerns about generalizability, as much of the available data originates from single-center studies,^30^ partly owing to the fact that NTM-D is not a reportable disease.^16^ In addition, some studies did not clearly distinguish between NTM infection and NTM disease, further complicating interpretation.^31^ NTM-D frequently presents as a late-onset complication, yet several studies have relied on short observation periods, sometimes limited to just 1 year after transplant.^21,32^ A systematic review reported a median time to NTM infection of 37 months (range, 3 days to 252 months) after renal transplant,^17^ whereas an observational study of 82 SOTRs with NTM-D found a mean time to diagnosis of 48 months (range, 10 days to 269 months).^19^ These findings underscore the need for extended follow-up to accurately assess the incidence and burden of posttransplant NTM-D.

NTM-D incidence among SOTRs varies by allograft type, with previous estimates ranging from 0.16% to 0.38% in kidney, 0.24% to 2.8% in heart, and 0.46% to 9.0% in lung allograft recipients.^16,17,18,19,33,34^ In our cohort, cumulative NTM-D incidence exceeded these prior single-center studies^23,32^ (22.59% in lung, 3.59% in heart, 0.59% in kidney, and 0.55% in liver transplant recipients), although differences in follow-up duration may limit direct comparisons. Overall, 2.92% of SOTRs developed NTM-D, approximately 30 times greater than the 0.10% incidence observed in the general population. Compared with matched nontransplant controls for each organ type, recipients of lung, heart, kidney, and liver allografts experienced significantly higher NTM-D rates, supporting a potential role of immunosuppression in increasing susceptibility.

Lung transplant recipients are at especially high risk for NTM-D due to direct environmental exposure of the lung allograft, frequent pulmonary complications, and, in some cases, a residual diseased native lung after unilateral transplant.^34^ Considering surveillance bronchoscopy in lung transplants, this risk may be overestimated in some studies. In a single-center case-control study by Longworth et al^35^ with an observation period of 7 years after transplant, including 34 patients with NTM-D, only lung transplant recipients were found to be at NTM-D risk, suggesting limited relevance of NTM-D to nonlung SOTRs. In contrast, our population-based cohort with extended follow-up demonstrated significantly increased NTM-D risk in both lung and nonlung SOTRs compared with matched controls, underscoring broader applicability across transplant types.

Chronic lung diseases (eg, bronchiectasis, bronchiolitis, chronic obstructive pulmonary disease, or interstitial lung disease) are known risk factors for NTM-D and were significantly associated with NTM-D in both lung and nonlung SOTRs in our study.^24,36^ We also found a significant association between rheumatoid arthritis and NTM-D, consistent with a prior provincial study showing a greater than 40-fold increased risk in patients with rheumatoid arthritis.^37^ Immigration status was also associated with NTM-D risk in lung transplant recipients, likely reflecting prior exposure in countries of origin.^38,39^ Despite these associations, the underlying pathophysiology of NTM-D remains incompletely understood.

Some studies have suggested that NTM-D in SOTRs is not necessarily associated with increased mortality,^19,20,21^ but more recent evidence challenges this notion. A recent case-control study comparing 85 SOTRs with NTM pulmonary disease with 190 SOTRs without found that NTM pulmonary disease was associated with an increased risk of 1-year mortality.^22^ In our cohort, multivariable analysis adjusted for key comorbidities showed that NTM-D was independently associated with a more than 2-fold increase in long-term mortality among SOTRs. Covariates previously shown to be associated with mortality after NTM-D diagnosis (eg, chronic heart or lung disease, history of stroke, diabetes, rheumatoid arthritis, and frailty score) were included in the model and were themselves significantly associated with increased mortality risk.^37,40^

Although residual confounding cannot be entirely excluded—raising the possibility that NTM-D may reflect underlying frailty rather than a direct cause of death—our findings suggest that NTM-D contributes to cumulative disease burden, clinical deterioration, and heightened mortality. Notably, in stratified analysis, NTM-D caused by MAC, RGM, and *M xenopi* was associated with increased mortality, whereas *M lentiflavum* was not. In the stratified, adjusted analysis of long-term mortality, the association between mortality and NTM-D caused by MAC or RGM remained statistically significant in both lung and nonlung SOTRs. In contrast, the association between mortality and *M xenopi* was no longer statistically significant in either transplant group. This finding is particularly noteworthy in the context of recent data from Ontario demonstrating increasing incidence of NTM-D due to MAC and decreasing incidence of *M xenopi*.^10^ Although studies in the general population have reported substantially higher mortality associated with RGM compared with MAC,^9^ our findings suggest that among SOTRs, NTM-D due to MAC and RGM is associated with comparable mortality risk.

### Strengths and Limitations

The main strength of our study is its large-scale, population-based cohort of over 12 000 SOTRs, enhancing generalizability. The nearly 2-decade study interval enabled us to capture both early-onset and late-onset NTM-D, assess long-term outcomes, and include diverse transplant types and comorbidity profiles, thereby supporting the external validity of our findings.

Our study also has several limitations. First, we lacked individual-level data such as clinical information, imaging studies, rejection episodes, and immunosuppression. Second, NTM-D treatment data were unavailable in administrative databases, which prevented an evaluation of the treatment’s impact. Given the prolonged and complex nature of NTM-D therapy, future prospective studies are needed. Third, we did not apply a fixed latency period or evaluate the association between NTM-D occurring before and after the index date, considering routine treatment of pretransplant NTM infections in lung transplant candidates, the lack of screening for those who receive nonlung transplants, and the absence of association between pretransplant and posttransplant NTM-D in patients with nonpulmonary NTM-D, which may arise shortly after transplantation. Although Friedman et al^41^ found an association between pretransplant and posttransplant NTM-D in lung recipients, this finding was not consistently replicated in other studies.^18,21,42^ Fourth, the higher mortality in SOTRs likely reflects posttransplant complications and underlying illness not fully captured by administrative data.

## Conclusions

In this cohort study of SOTRs and the general population, SOTRs had a significantly higher risk of NTM-D. This association was evident in both lung and nonlung allograft recipients and persisted even after accounting for comorbidities known to be associated with NTM-D risk. Furthermore, NTM-D was associated with markedly increased mortality risk in lung and nonlung SOTRs, particularly when caused by MAC or RGM.

## Notes

Supplement 1. **eFigure**. Study flowchart **eTable 1.** Data sources used for comorbidities and medical conditions **eTable 2.** Total number of NTM isolates detected in the cohort, after exclusion of single sputum culture results **eTable 3.** NTM-PD risk in the non-lung and lung transplant SOTRs compared to the general population, unadjusted analysis **eTable 4.** Adjusted Cox proportional hazard model estimating the one-year and the long-term mortality risk in SOTRs considering NTM-D **eTable 5.** Adjusted Cox proportional hazard model estimating the one-year and the long-term mortality risk in SOTRs, considering five categories of NTM-D
eReference

Supplement 2.
Data Sharing Statement
