# Supplementary material for: Nontuberculous Mycobacterial Disease in Solid-Organ Transplant Recipients and the General Population
Source: JAMA Netw Open. 2025 Sep 12;8(9):e2531563. doi: 10.1001/jamanetworkopen.2025.31563 (PMC12432629; doi:10.1001/jamanetworkopen.2025.31563)
Supplement: Supplement 1. — eFigure. Study flowchart eTable 1. Data sources used for comorbidities and medical conditions eTable 2. Total number of NTM isolates detected in the cohort, after exclusion of single sputum culture results eTable 3. NTM-PD risk in the non-lung and lung transplant SOTRs compared to the general population, unadjusted analysis eTable 4. Adjusted Cox proportional hazard model estimating the one-year and the long-term mortality risk in SOTRs considering NTM-D eTable 5. Adjusted Cox proportional hazard model estimating the one-year and the long-term mortality risk in SOTRs, considering five categories of NTM-D eReference [file jamanetwopen-e2531563-s001.pdf]

Supplemental Online Content

Hosseini-Moghaddam SM, Fridman D, Drover SSM, et al. Nontuberculous mycobacterial disease in solid-organ transplant recipients and the general population. *JAMA Netw Open*. 2025;8(9):e2531563. doi:10.1001/jamanetworkopen.2025.31563

eFigure. Study flowchart
eTable 1. Data sources used for comorbidities and medical conditions
eTable 2. Total number of NTM isolates detected in the cohort, after exclusion of single sputum culture results
eTable 3. NTM-PD risk in the non-lung and lung transplant SOTRs compared to the general population, unadjusted analysis
eTable 4. Adjusted Cox proportional hazard model estimating the one-year and the long-term mortality risk in SOTRs considering NTM-D
eTable 5. Adjusted Cox proportional hazard model estimating the one-year and the long-term mortality risk in SOTRs, considering five categories of NTM-D
eReference

This supplemental material has been provided by the authors to give readers additional information about their work.

© 2025 Hosseini-Moghaddam SM et al. *JAMA Network Open*.

eFigure. Study flowchart

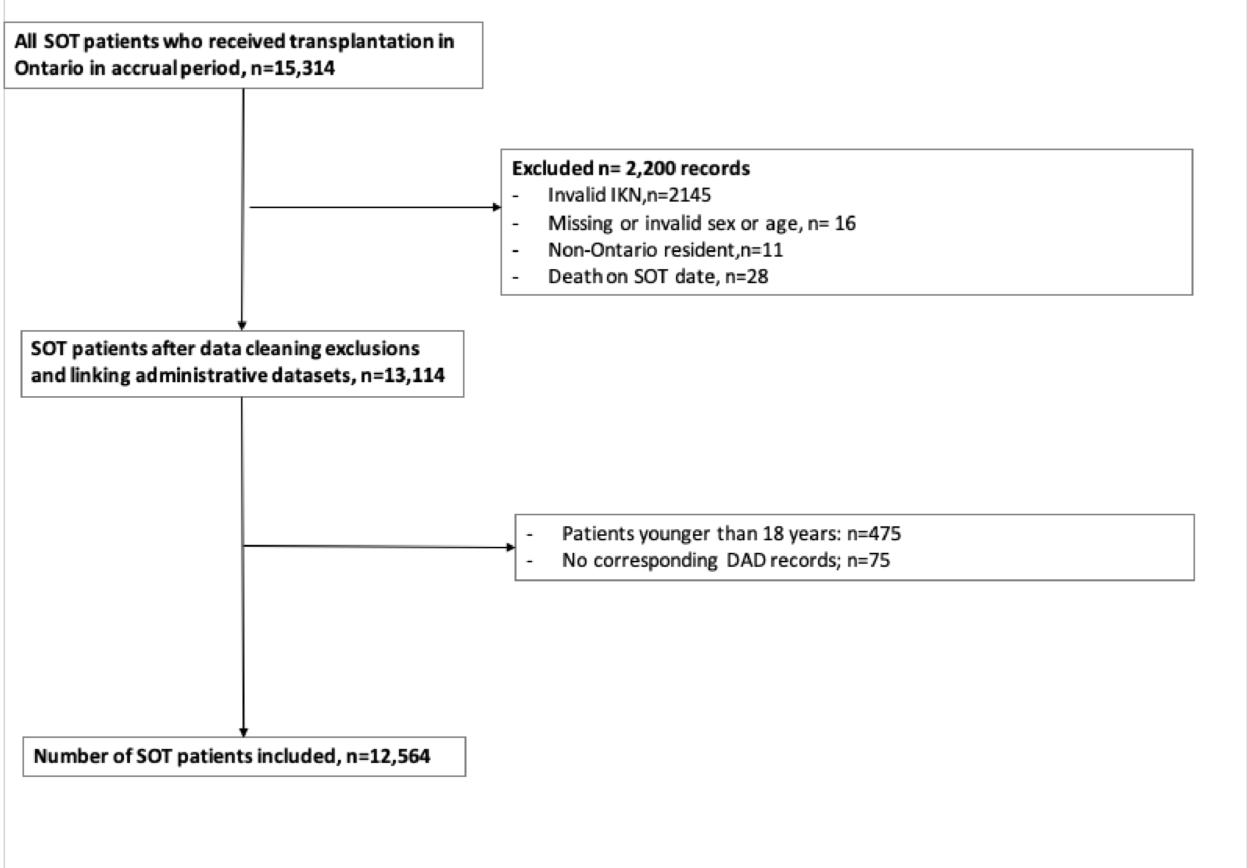

DAD: Discharge Abstract Database; IKN refers to the ICES Key Number, a unique, confidential identifier assigned to each individual in Ontario within ICES datasets.

© 2025 Hosseini-Moghaddam SM et al. *JAMA Network Open*.

eTable 1. Data sources used for comorbidities and medical conditions

Medical Condition	Definition
Asthma<br>(ICES cohort)	Asthma database was used to identify patients with asthma, based on 2 or more ambulatory care visits and/or 1 or more hospitalizations.<br><br><u>Ontario Health Insurance Plan (OHIP)</u><br>OHIP diagnostic code: 493<br><br><u>Canadian Institute for Health Information- Discharge Abstract Database (CIHI-DAD)</u><br>ICD-9 diagnostic code: 493<br>ICD-10 diagnostic codes: J45, J46
Chronic obstructive pulmonary disease (COPD)<br>(ICES specific cohort)	COPD database was used to identify patients with COPD, based on 1 or more ambulatory care visits and/or 1 or more hospitalizations. Algorithm to identify COPD patients were only validated in those ages 35 and over.(2)<br><br><u>OHIP</u><br>OHIP diagnostic codes: 491, 492, 496<br><br><u>CIHI-DAD</u><br>ICD-9 diagnostic codes: 491, 492, 496<br>ICD-10 diagnostic codes: J41, J42, J43, J44
Diabetes<br>(ICES cohort)	Ontario Diabetes Database (ODD) was used to identify patients with diabetes, based on 2 OHIP diagnostic codes or 1 OHIP service code or 1 CIHI admission within 2 years.(3)<br><br><u>OHIP</u><br>OHIP diagnostic code: 250<br>OHIP service codes: Q040, K029, K030, K045, K046<br><br><u>CIHI-DAD, CIHI-Same Day Surgery (SDS)</u><br>ICD-9 diagnostic code: 250<br>ICD-10 diagnostic codes: E10, E11, E13, E14
Active cancer	i) Had any of the following treatments in the past 6 months:<br>- Cancer surgery – see “SurgeryIn6Month_CCI_for Active Cancer concept.xlsx” Canadian Classification of Health Interventions (CCI) codes in DAD<br>- Radiation - using %getnacrs where source = Cancer and DX10CODE1 = Z510<br>- Chemotherapy - using %getnacrs where source = Cancer and DX10CODE1 = Z511 or Z512<br>AND any evidence of cancer diagnosis in Ontario Cancer Registry (OCR) prior to last treatment date (surgery, radiation and chemotherapy).<br><br>II) If not I) (i.e., no recent treatment for cancer), if they had a recent diagnosis – cancer diagnosis in OCR within the past year before index
Chronic kidney disease (CKD)<br>(DAD, NACRS, OHIP)	• CKD diagnosis code in DAD, NACRS, OHIP in 5 years <u>or</u><br>• At least 1 dialysis code in each of the 3 months prior to index<br>• Diagnosis and procedure codes found in concept dictionary

© 2025 Hosseini-Moghaddam SM et al. *JAMA Network Open*.

Medical Condition	Definition
	OHIP<br>OHIP diagnostic codes: 403, 585<br><br>CIHI-National Ambulatory Care Reporting System (NACRS), CIHI-DAD<br>ICD-10 diagnostic codes: E102, E112, E132, E142, I12, I13, N08, N18, N19
	Patients who were on chronic dialysis (6) in the year before index date, identified as those with at least 2 of any of the following codes in OHIP, CIHI-DAD, or CIHI-SDS separated by at least 90 days, but less than 150 days<br><br>OHIP<br>OHIP service codes: R849, G323, G325, G326, G860, G862, G865 G863, G866, G330, G331, G332, G333, G861, G082, G083, G085, G090, G091, G092, G093, G094, G095, G096, G294, G295, G864, H540, H740<br><br>CIHI-DAD, CIHI-SDS<br>CCI procedure codes: 5195, 6698<br>Canadian Classification of Diagnostic, Therapeutic and Surgical Procedures (CCP) code: 1PZ21
Advanced liver disease (Cirrhosis or Decompensated Cirrhosis)	See Lapointe-Shaw 2018 "Identifying cirrhosis…"<br><br>Any history of: look back to start of data<br><br>Cirrhosis Algorithm 9<br>2+ Physician Visit (dxcode 571) or<br>1+ Hospital Diagnosis CIRRHOSIS<br>ICD-9 : 456.1, 571.2, 571.5<br>ICD-10: I85.9, I98.2, K70.3,K71.7, K74.6<br><br>Decompensated Cirrhosis Algorithm 5<br>1+ Physician Visit CIRRHOSIS (dxcode 571)<br>and (1+ Hospital Diagnosis or 1+ Procedure)<br>ICD-9: 456.0, 456.2, 572.2, 572.3, 572.4, 782.4, 789.5<br>ICD-10: I85.0, I86.4, I98.20, I98.3, K721, K729, K76.6, K76.7, R17, R18<br>CCI: 1.NA.13.BA-FA, 1.NA.13.BA-X7, 1.NA.13.BA-BD, 1.KQ.76GP-NR, 1.OT.52.HA<br>CCP: 1006, 6691<br>OHIP: J057, Z591
Inflammatory Bowel Disease	Ontario Crohn's and Colitis Cohort (OCCC) definition
Dementia/Frailty	Dementia (ICES cohort):<br>1 hospitalization for dementia and/or 3 ambulatory visits for dementia, each separated by at least 30 days, within 2 years<br>and/or 1 prescription from ODB (9)<br><br>OHIP<br>OHIP diagnostic codes: 290, 331<br><br>CIHI-DAD, CIHI-SDS<br>ICD-9 diagnostic codes: 0461, 290.0, 290.1, 290.2, 290.3, 290.4, 294, 331.0, 331.1, 331.5

© 2025 Hosseini-Moghaddam SM et al. *JAMA Network Open*.

Medical Condition	Definition
	ICD-10 diagnostic codes: F00, F01, F02, F03, G30<br><br><u>Ontario Drub Benefit plan (ODB)</u><br>1 prescription for a cholinesterase inhibitor<br><br><u>Frailty:</u><br>Hospital Frailty Risk Score - "Summarized" frailty score – Pull all DAD hospitalizations in the 5-years before index, and use %hospfrailty macro to determine "summarized" frailty score per ICES Key Number (IKN). (Gilbert 2018, Lancet)<br>NOTE: Codes used in this risk score also include frailty related conditions such as dementia and other chronic conditions that are reported separately.
Hypertension	HYPER database
History of congestive heart failure (CHF) (ICES cohort)	CHF database was used to identify patients with CHF, based on 1 CIHI NACRS, CIHI-DAD, CIHI-SDS, or OHIP claim and a second claim (from either) in 1 year. The CHF database is limited to those 40 years of age or older.(11)<br><br><u>OHIP</u><br>OHIP diagnostic code: 428<br><br><u>CIHI-DAD, CIHI-SDS</u><br>ICD-9 diagnostic code: 428<br>ICD-10 diagnostic codes: I500, I501, I509
History of transient ischemic attack (TIA) or Acute Ischemic Stroke	<u>Transient Ischemic Attack:</u><br>CIHI-DAD and CIHI-NACRS were used to identify patients with a history of a transient ischemic attack, based on at least 1 hospitalization or ED visit with a diagnosis coded with one of the following codes:<br><br>ICD-9 diagnostic codes: 435, 3623<br>ICD-10 diagnostic codes: G450, G451, G452, G453, G458, G459, H340<br><br><u>Acute Ischemic Stroke (12):</u><br>CIHI-DAD was used to identify patients with a history of acute ischemic stroke, based on at least 1 hospitalization with a main diagnosis coded with one of the following codes:<br><br>ICD-9 diagnostic codes: 43301, 43311 43321 43331 43381 43391 434, 436<br>ICD-10 diagnostic codes: I63, I64, H34.1
History of cardiac ischemia	Cardiac ischemic disease (DAD, SDS): Any comorbidity in the past 5 years (DAD, any diagnosis field) or history of procedure in past 20 years (DAD, SDS):<br><br>Comorbidity (DAD, any dx, 5-year lookback):<br>Angina: ICD-10 diagnostic codes: I20<br>ICD-9: 413<br><br>Chronic Ischemic Heart Disease: ICD-10 diagnostic codes: I25

© 2025 Hosseini-Moghaddam SM et al. *JAMA Network Open*.

Medical Condition	Definition
	ICD-9: 4140, 4148, 4149<br><br>Myocardial infarction: ICD-10 diagnostic codes: I21, I22<br>ICD-9: 410, 411, 412<br><br>Procedure (DAD & SDS, 20 year lookback):<br>Coronary Artery Bypass Grafting:<br>CCI procedure codes: 1IJ76<br>CCP procedure codes: 481<br><br>Percutaneous Coronary Intervention:<br>CCI procedure codes: 1IJ50, 1IJ5, 1IJ57<br>CCP procedure codes: 4802, 4803
Immigration	Immigration, Refugees and Citizenship Canada Permanent Resident (IRCC-PR) database
Death	RPDB

© 2025 Hosseini-Moghaddam SM et al. *JAMA Network Open*.

eTable 2. Total number of NTM isolates detected in the cohort, after exclusion of single sputum culture results

<table>
<tr><th></th><th>SOTR</th><th>Control</th></tr>
<tr><td>Mycobacterium Avium complex</td><td>245</td><td>85</td></tr>
<tr><td>Mycobacterium xenopi</td><td>73</td><td>16</td></tr>
<tr><td>RGM</td><td>56</td><td>13</td></tr>
<tr><td>Mycobacterium lentiflavum</td><td>24</td><td rowspan="2">13</td></tr>
<tr><td>Other NTMs[A]</td><td>7</td></tr>
<tr><td>Total</td><td>405</td><td>127</td></tr>
</table>

A: Other NTM includes *M. farcinogenes, M. arupense, M. longobardum, M. marinum, M. senegalense, M. terrae complex, M. franklinii, M. genavense, M. goodie, M. haemophilum, M. ilatzerense, M. immunogenum, M. intermedium, M. kubicae, M. paragordonae, M. parascrofulaceum, M. porcinum, M. scrofulaceum, M. szulgai*

eTable 3. NTM-PD risk in the non-lung and lung transplant SOTRs compared to the general population, unadjusted analysis

Organ type	HR(95% CI)
Non-lung transplant	10.93 (8.0-14.93)
Lung transplant	281.57 (149.87-529.01)

© 2025 Hosseini-Moghaddam SM et al. *JAMA Network Open*.

eTable 4. Adjusted Cox proportional hazard model estimating the one-year and the long-term mortality risk in SOTRs considering NTM-D

Variable	One-year mortality<br><br>Adjusted HR(95% CI)	Long-term mortality<br><br>Adjusted HR(95% CI)
Age	1.02 (1.01-1.03)	1.04 (1.03-1.04)
Sex (female vs male)	1.00 (0.86-1.17)	0.91 (0.86-0.98)
NTM-D	1.29 (0.83-2.01)	2.33 (2.00-2.71)
Chronic kidney disease	0.46 (0.38-0.55)	0.69 (0.63-0.76)
Chronic heart disease	1.67 (1.41-1.97)	1.32 (1.23-1.42)
Advanced liver disease	1.12 (0.93-1.35)	0.83 (0.76-0.91)
Chronic lung disease	1.23 (1.05-1.44)	1.36 (1.27-1.46)
History of stroke, transient ischemic attack	0.77 (0.53-1.12)	1.26 (1.12-1.43)
Hypertension	0.67 (0.56-0.80)	0.84 (0.77-0.92)
Diabetes	1.16 (1.00-1.35)	1.35 (1.26-1.44)
Inflammatory bowel disease	1.89 (1.00-3.56)	1.71 (1.17-2.48)
Rheumatoid arthritis	1.41 (0.96-2.08)	1.20 (1.00-1.44)
Dementia	0.98 (0.31-3.07)	0.91 (0.56-1.47)
Frailty Risk Score[A]	1.03 (1.01-1.04)	1.03 (1.02-1.04)
Rural residential status	1.09 (0.86-1.39)	0.98 (0.89-1.08)
Immigrant	1.11 (0.89-1.38)	0.78 (0.70-0.87)

A: Frailty Risk Score was calculated using ICD-10 codes of the health administrative data and the approach adapted from Gilbert et al.(1) A numerical score is determined by the number of relevant *ICD-10* codes from an individual's prior hospitalization.

© 2025 Hosseini-Moghaddam SM et al. *JAMA Network Open*.

eTable 5. Adjusted Cox proportional hazard model estimating the one-year and the long-term mortality risk in SOTRs, considering five categories of NTM-D.

Variable	One-year mortality<br>Adjusted HR(95% CI)	Long-term mortality<br>Adjusted HR(95% CI)
Age	1.02 (1.01 -1.03)	1.04 (1.03-1.04)
Sex, (female vs male)	1.00 (0.86 -1.17)	0.91 (0.85-0.98)
MAC [A]	1.46 (0.88-2.40)	2.28 (1.89-2.73)
RGM[A]	2.30 (0.85 -6.24)	3.00 (2.14-4.19)
M. xenopi	0.53 (0.13 -2.16)	1.31 (1.01-1.91)
M. lentiflavum	1.84 (0.58 -5.80)	1.63 (0.90-2.96)
Other NTM	0.00	5.11 (1.91-13.67)
Chronic kidney disease	0.46 (0.38 -0.56)	0.68 (0.62-0.75)
Chronic heart disease	1.67 (1.41 -1.97)	1.32 (1.22-1.41)
Advanced liver disease	1.12 (0.93 -1.35)	0.82 (0.75-0.90)
Chronic lung disease	1.22 (1.04 -1.44)	1.36 (1.26-1.45)
History of stroke, transient ischemic attack	0.77 (0.53 -1.12)	1.26 (1.11-1.42)
Hypertension	0.67 (0.56 -0.80)	0.83 (0.76-0.91)
Diabetes	1.16 (0.99 -1.34)	1.34 (1.25-1.43)
Inflammatory bowel disease	1.90 (1.01-3.57)	1.70 (1.17-2.47)
Rheumatoid arthritis	1.41 (0.95 -2.07)	1.19 (0.99-1.43)
Dementia	0.99 (0.32 -3.08)	0.90 (0.56-1.46)
Frailty Risk Score	1.03 (1.01 -1.04)	1.02 (1.02-1.03)

© 2025 Hosseini-Moghaddam SM et al. *JAMA Network Open*.

Immigration	1.11 (0.89 -1.38)	0.77 (0.69-0.86)
Rural residential status	1.09 (0.86-1.39)	0.98 (0.89-1.08)

A: *Mycobacterium avium* complex (MAC) included *M. avium, M. intracellulare group, M. chimaera,* and *M. marseillense*; and rapidly growing *mycobacteria* (RGM) included *M. fortuitum* group, *M. chelonae, M. abscessus, M. mucogenicum-phocaicum* group, and *M. neoaurum.*

eReference

1. Gilbert T, Neuburger J, Kraindler J, Keeble E, Smith P, Ariti C, et al. Development and validation of a Hospital Frailty Risk Score focusing on older people in acute care settings using electronic hospital records: an observational study. Lancet (London, England) [Internet]. 2018 May 5 [cited 2025 Feb 11];391(10132):1775–82. Available from: https://pubmed.ncbi.nlm.nih.gov/29706364/

© 2025 Hosseini-Moghaddam SM et al. *JAMA Network Open*.
