# Supplementary material for: Nontuberculous Mycobacterial Disease in Solid-Organ Transplant Recipients and the General Population
Source: JAMA Netw Open. 2025 Sep 12;8(9):e2531563. doi: 10.1001/jamanetworkopen.2025.31563 (PMC12432629; doi:10.1001/jamanetworkopen.2025.31563)
Supplement: Supplement 2. — Data Sharing Statement [file jamanetwopen-e2531563-s002.pdf]

Data Sharing Statement

Hosseini-Moghaddam. Nontuberculous Mycobacterial Disease in Solid-Organ Transplant Recipients and the General Population. *JAMA Netw Open*. Published September 12, 2025. doi:10.1001/jamanetworkopen.2025.31563

Data
Data available: Yes
Data types: Deidentified participant data
How to access data: The data availability should be confirmed with ICES.
When available: With publication

Supporting Documents
Document types: None

Additional Information
Who can access the data: ICES should approve availability of statistical/analytic codes.
Types of analyses: For a specified purpose
Mechanisms of data availability: After approval of a proposal
